# Supplementary material for: WordCommentsAnalyzer: A windows software tool for qualitative research
Source: F1000Res. 2018 Sep 4;7:536. Originally published 2018 May 3. [Version 2] doi: 10.12688/f1000research.14819.2 (PMC6137411; doi:10.12688/f1000research.14819.2)
Supplement: Supplementary file 3 [file f1000research-7-17614-s0002.tgz › b2a79496-4ada-47c4-9682-a99ce867836d.zip!/Eight Iranian Health Journals/Journal of Education and Community Health_3.docx]

© Journal of Education and Community Health. This work is licensed under CC BY-NC 4.0 (https://creativecommons.org/licenses/by-nc/4.0/).

Volume & Issue: دوره 4 - شماره 2

Title: نقش ابزارهای اشتراک نتایج پژوهش های سلامت در ارتقای سواد سلامت جامعه

http://jech.umsha.ac.ir/article-1-346-fa.html

سردبیر محترم شاخص‌های علم‌سنجی متنوعی برای ارزیابی کیفیت مجلات علمی تبیین و معرفی شده‌اند که در ‌این‌ میان، می‌توان به معروف‌ترین آن؛ یعنی ضریب تأثیر (Impact Factor) اشاره نمود. علاوه‌بر‌این، شاخص‌هایی مانند g index، Cite Score و نوع نمایه مجلات به‌عنوان شاخص‌های علم‌سنجی مطرح می‌باشند.  بیشتر این شاخص‌ها در رابطه با کیفیت مجلات مصداق دارند و از‌ آنجایی که رتبه‌بندی و ارزیابی کیفیت مقالات چاپ‌شده در مجلات حائز ‌اهمیت فراوانی است، امروزه شاخص‌هایی در سطح مقاله که از آن‌ها به‌عنوانArticle Level Metric یاد می‌شود، معرفی شده و مورد‌ توجه متخصصان قرار گرفته‌اند؛ به‌عنوان‌ مثال، تعداد استنادات به یک مقاله مبنای کیفیت آن قرار می‌گیرد. با‌ این ‌وجود تعداد استناد به یک مقاله، نشانه اثرگذاری آن در سطح علمی می‌باشد؛ اما حاکی از اثرگذاری اجتماعی مقاله در حوزه مربوطه نخواهد بود. با توجه به اهمیت کاربست نتایج حاصل از مقالات و ارتقای سواد سلامت جامعه، وجود شاخص‌هایی که اثرگذاری اجتماعی انتشار مقاله به زبان قابل‌فهم برای عموم جامعه را معرفی نماید، یک ضرورت می‌باشد. در این ارتباط، پایگاه‌های اطلاعاتی از راه‌کارهای متنوعی برای انجام این رسالت بهره برده‌اند و امروزه ابزارهای اشتراک مقاله در کنار فایل مقالات، در دسترس خوانندگان می‌باشد. شایان‌ ذکر است که پایگاه اطلاعاتی PubMed، ابزار PubMed Commons و Scopus، ابزار PlumX Metrics را بدین‌منظور راه‌اندازی کرده‌اند. این ابزار در پایگاه Scopus تعداد دفعات مشاهده مقاله، به‌ اشتراک‌گذاری محتوای مقاله در شبکه‌های اجتماعی از جمله Twitter، Facebook و Google+ و نیز تعداد دفعات خوانده‌شدن مقاله و یا دانلود آن درMendeley  و Science Direct را در اختیار کاربران قرار می‌دهند. از ‌این ‌رو، در راستای تحقق رسالت نظام سلامت در ارتقای سواد سلامت جامعه پیشنهاد می‌شود که در مورد ابزارهای چگونگی به اشتراک‌گذاری پیام‌های پژوهش توسط فعالان در زمینه آموزش بهداشت و ارتقای سلامت به عموم جامعه و خوانندگان اطلاع‌رسانی شود.

Title: اثربخشی زوج‌درمانی مبتنی ‌بر نظریه انتخاب بر تعهد اجتماعی زوجین

http://jech.umsha.ac.ir/article-1-298-fa.html

سابقه و هدف: وفاداری به همسر و تعهد نسبت به ازدواج و خانواده، یکی از مهم‌ترین فاکتورهای تضمین‌کننده ماندگاری ازدواج و سلامت نهاد خانواده است که در مطالعات نوین خانواده و ازدواج مورد توجه جدی پژوهشگران قرار گرفته است. در این ‌راستا، هدف از پژوهش حاضر تعیین تأثیر زوج‌‌درمانی مبتنی‌ بر نظریه انتخاب بر تعهد اجتماعی زوجین بود. مواد و روش‌‌ها: پژوهش حاضر یک مطالعه نیمه‌تجربی با طرح پیش‌آزمون- پس‌آزمون همراه با گروه کنترل بود که در بین زوجین داوطلب مراجعه‌کننده به مراکز مشاوره و خدمات روان‌شناختی شهر اصفهان در سال 1394 اجرا گردید. نمونه آماری‌ پژوهش را 32 زوج ناسازگار تشکیل دادند که به ‌روش نمونه‌گیری در دسترس انتخاب شدند و به‌صورت تصادفی در گروه‌های آزمون (16 زوج) و کنترل (‌16 زوج) قرار گرفتند. اعضای گروه آزمون در 9 جلسه زوج‌درمانی گروهی طی مدت‌زمان 3 ماه شرکت نموده و مهارت‌های زندگی خانوادگی مربوط به نظریه انتخاب را فراگرفتند. شایان‌ ذکر است که متغیر وابسته، تعهد اجتماعی همسران بود که توسط پرسشنامه ابعاد تعهد Adams و Jones (Dimensions of Commitment Inventory DCI:) ارزیابی گردید. داده‌ها نیز پس از جمع‌آوری به‌وسیله تحلیل کواریانس چند‌متغیره با استفاده از نرم‌افزار آماری SPSS 20  تحلیل شدند. یافته‌ها: یافته‌ها نشان داد که زوج‌درمانی مبتنی ‌بر نظریه انتخاب، تعهد اجتماعی زوجین گروه آزمون را در مقایسه با گروه کنترل به‌طور معناداری در مرحله پس‌آزمون افزایش داده است (0.001>P). نتیجه‌گیری: با توجه به نتایج حاصل از این مطالعه، زوج‌درمانی گروهی مبتنی ‌بر نظریه انتخاب، راه‌کار مناسبی برای توسعه و تحکیم سطح وفاداری زوجین نسبت به همسر، ازدواج و خانواده می‌باشد و می‌تواند نقش مؤثری را در کاهش و پیشگیری از معضلات و آسیب‌های فراگیر حوزه خانواده و ازدواج از قبیل طلاق و عهدشکنی زناشویی ایفا نماید.

Title: سواد سلامت و عوامل مرتبط با آن در دانشجویان دانشگاه علوم ‌پزشکی همدان

http://jech.umsha.ac.ir/article-1-332-fa.html

سابقه و هدف: سواد سلامت در افراد تأثیر بالقوه‌ای بر پیشبرد سلامتی و کیفیت زندگی آن‌ها دارد و به ‌همان نسبت موجب کاهش هزینه‌های مربوط به مراقبت‌های بهداشتی می‌شود؛ از این ‌رو، با توجه به اهمیت سواد سلامت و نقش آن در سلامت افراد، پژوهش حاضر با هدف تعیین سطح سواد سلامت و عوامل مرتبط با آن در دانشجویان دانشگاه علوم‌ پزشکی همدان صورت گرفت. مواد و روش‌‌ها: این مطالعه مقطعی در سال 1395 روی 386 نفر از دانشجویان مشغول به تحصیل در رشته‌های مختلف دانشگاه علوم ‌پزشکی همدان که با روش نمونه‌گیری چند‌مرحله‌ای انتخاب شدند، انجام گرفت. داده‌ها به‌ روش خودگزارش‌دهی و به‌وسیله پرسشنامه سواد سلامت بزرگسالان ایرانی (HELIA: Health Literacy for Iranian Adults) جمع‌آوری شدند و با استفاده از نرم‌افزار SPSS 21 و بهره‌گیری از آمار توصیفی و تحلیلی شامل آزمون‌های Chi-Square، T مستقل و ANOVA تجزیه‌ و تحلیل گردیدند. یافته‌ها: 50.5 درصد از دانشجویان سواد سلامت کافی داشتند و 3.9 درصد دارای سواد سلامت ناکافی بودند. همچنین، آن‌ها بیشترین اطلاعات مربوط به سواد سلامت خود را از طریق اینترنت کسب می‌کردند. براساس نتایج، بین متغیرهای سن، جنسیت، تأهل، دانشکده محل تحصیل و نوع منابع کسب اطلاعات دانشجویان با ابعاد سواد سلامت ارتباط معناداری به‌دست آمد (0.05>P). نتیجه‌گیری: با توجه به سطح سواد سلامت و متغیرهای جمعیت‌شناختی اثرگذار، لازم است مداخلات مؤثری به‌منظور توانمندسازی دانشجویان در زمینه ارتقای سواد سلامت طراحی و اجرا گردد. همچنین، لازم است روش‌های جدیدتری مانند آموزش تحت‌وب به‌منظور کسب اطلاعات بهداشتی بیشتر در اختیار دانشجویان قرار داده شود.

Title: پیش‌بینی رفتار پرخطر در افراد مبتلا به HIV براساس عملکرد خانوادگی: با درنظرگرفتن متغیرهای میانجی سبک زندگی و تصمیم‌گیری پرخطر

http://jech.umsha.ac.ir/article-1-277-fa.html

سابقه و هدف: شیوع رفتارهای پرخطر در جمعیت مبتلا به HIV بیشتر از جمعیت عمومی است که این امر ناشی از عواملی چون عوامل زیستی، خانوادگی و اجتماعی، تأثیر گروه هم‌سالان و رسانه و داشتن سبک زندگی می‌باشد. یکی از پیش‌بین‌های مهم رفتارهای پرخطر در میان این جمعیت، عملکرد خانوادگی ضعیف افراد مبتلا است. در این‌ راستا هدف از پژوهش حاضر، بررسی نقش کارکرد خانوادگی در پیش‌بینی تصمیم‌گیری پرخطر در افراد مبتلا به HIV بود که با درنظرگرفتن نقش میانجی متغیرهای سبک زندگی و تصمیم‌گیری پرخطر انجام پذیرفت. مواد و روش‌‌ها: پژوهش حاضر از نوع مطالعات توصیفی- همبستگی می‌باشد که روی 147 فرد مبتلا به HIV صورت گرفته و در آن از روش نمونه‌گیری در دسترس استفاده شده است. آزمودنی‌ها در این پژوهش به پرسشنامه سبک زندگی ارتقا‌دهنده سلامت ـ2 (Health Promoting Lifestyle Profile-2: HPLP-2)، مقیاس انسجام و انعطاف‌پذیری خانواده (Family Adaptability and Cohesion Scale IV :FACES-IV)، ابزار سنجش رفتار پرخطر در موقعیت‌های اجتماعی و آزمون خطرپذیری بادکنکی (Balloon Analogue Risk Task :BART) پاسخ دادند. لازم ‌به ‌ذکر است که تحلیل داده‌ها با استفاده از روش مدل‌یابی معادلات ساختاری و نرم‌افزار LISREL 8.8 صورت گرفت. یافته‌ها: نتایج این مطالعه نشان داد که همبستگی منفی و معناداری بین کارکرد خانوادگی و رفتارهای پرخطر وجود دارد که در این ‌میان، کارکرد خانوادگی به‌طور مستقیم و غیرمستقیم توسط دو متغیر میانجی سبک زندگی و تصمیم‌گیری پرخطر بر رفتارهای پرخطر تأثیر می‌گذارد. نتیجه‌گیری: نتایج مطالعه حاضر نشان داد که عملکرد خانوادگی به‌صورت مستقیم و غیرمستقیم ‌از طریق نقش میانجی تصمیم‌گیری پرخطر و‌ سبک زندگی با رفتار پرخطر رابطه دارد. در این‌ زمینه، پژوهش‌های آینده می‌بایست نقش عوامل خانوادگی را در رفتار پرخطر افراد مبتلا به HIV بیش‌تر مدنظر قرار دهند.

Title: ارتباط اضطراب، استرس و افسردگی با سلامت معنوی در بیماران سندرم حاد عروق کرونری

http://jech.umsha.ac.ir/article-1-271-fa.html

سابقه و هدف: اضطراب، استرس و افسردگی از علل بروز درد در قفسه سینه می‌باشند. از سوی‌ دیگر، سلامت معنوی از عوامل اصلی سازگاری با بیماری به‌شمار می‌رود. در این‌ راستا، پژوهش حاضر با هدف ارتباط اضطراب، استرس و افسردگی با سلامت معنوی در بیماران سندرم حاد عروق کرونر انجام شد. مواد و روش‌‌ها: در این پژوهش توصیفی- تحلیلی که روی 294 بیمار مبتلا به سندروم عروق کرونر در بیمارستان‌های آموزشی قم در سال 1393 صورت گرفت، داده‌ها با استفاده از پرسشنامه‌های اطلاعات زمینه‌ای و بالینی، افسردگی، اضطراب، استرس و سلامت معنوی جمع‌آوری گردید. تحلیل داده‌ها نیز با استفاده از نرم‌افزار SPSS 13 و آزمون‌های آماری شاخص‌های مرکزی، T مستقل و همبستگی پیرسون انجام شد. یافته‌ها: اضطراب و استرس با بعد سلامت وجودی و افسردگی با هر دو بعد سلامت وجودی و مذهبی همبستگی معناداری را نشان دادند. همچنین در افراد با سن بالا، میزان افسردگی و استرس بالاتر بود؛ اما سلامت وجودی کاهش داشت. از سوی‌ دیگر در افراد با سابقه بیماری مزمن، استرس، افسردگی و سلامت وجودی و مذهبی بیشتر بود. در افراد با سابقه مصرف داروی قلبی نیز افسردگی کمتر بود؛ اما استرس، اضطراب و سلامت وجودی و مذهبی بیشتری مشاهده شد. نتیجه‌گیری: بر‌اساس نتایج مشاهده شد که در میان مبتلایان به سندروم حاد عروق کرونری، افرادی که نمره سلامت معنوی بیشتری را کسب نمودند، اضطراب، استرس و افسردگی کمتری را تجربه می‌کردند؛ ازاین‌رو، پیشنهاد می‌‌شود پرستاران با حضور مشاوران دینی، برنامه‌های آموزشی خودمراقبتی را در بیمارستان‌ها و حتی پس از ترخیص با جدیت بیشتری پیگیری نمایند تا از این طریق بتوان به افزایش سلامت معنوی و کاهش اضطراب، استرس و افسردگی در آن‌ها کمک نمود. لازم ‌به ‌ذکر است که در این ‌میان، افراد بالاتر از 50 سال با سابقه بیماری مزمن و مصرف داروی قلبی باید بیشتر مورد توجه قرار گیرند.

Title: اثربخشی آموزش گروهی بهداشت بلوغ به مادران بر تعارضات والد- فرزندی مادران و کنترل هیجانات دختران نوجوان آن‌ها

http://jech.umsha.ac.ir/article-1-352-fa.html

سابقه و هدف: نوجوانی و بلوغ مرحله مهمی از رشد انسان است. بلوغ باعث تغییرات فیزیولوژیکی، اجتماعی و روان‌شناختی در نوجوانان می‌شود و این تغییرات بر ابعاد مختلف زندگی فرد از جمله روابط والد- فرزندی و کنترل عواطف نوجوانان تأثیر می‌گذارد. در این راستا پژوهش حاضر با هدف تعیین تأثیر آموزش گروهی بهداشت بلوغ به مادران بر تعارضات والد- فرزندی و کنترل هیجانات نوجوانان دختر انجام شد. مواد و روش‌‌ها: پژوهش حاضر یک مطالعه نیمه‌تجربی از نوع پیش‌آزمون- پس‌آزمون همراه با گروه کنترل بود. جامعه آماری پژوهش را کلیه مادران دانش‌آموزان دختر پایه هفتم منطقه 14 شهر تهران در سال تحصیلی 95-1394 تشکیل دادند. بدین‌منظور 30 مادر با استفاده از روش نمونه‌گیری خوشه‌ای بر حسب مناطق 20 گانه انتخاب شدند و به‌طور تصادفی به دو گروه آزمون و کنترل تقسیم گردیدند. آموزش گروهی بهداشت بلوغ طی 12 جلسه هفتگی و در مدت یک ساعت و نیم انجام شد. ابزارهای گردآوری اطلاعات شامل: پرسش‌نامه مشخصات جمعیت‌شناختی، پرسش‌نامه تعارض با والدین (Conflict to Parents) و مقیاس کنترل عواطف بود که قبل و بعد از مداخله آموزشی تکمیل گردید. شایان ذکر است که داده‌ها در نرم‌افزار 16  SPSS و با استفاده از روش تحلیل کواریانس تک متغیره تحلیل شدند. یافته‌ها: نتایج نشان داد که تفاوت معناداری در میانگین نمرات تعارض والد- فرزندی و تمامی خرده‌مقیاس‌های آن پس از مداخله آموزشی در گروه آزمون نسبت به گروه کنترل وجود دارد (0.05>P). همچنین نمرات میانگین کنترل عواطف و کل خرده‌مقیاس‌های آن در دختران آن‌ها پس از مداخله آموزشی در گروه آزمون در مقایسه با گروه کنترل کاهش نشان داد (0.05>P). نتیجه‌گیری: داشتن دانش و آگاهی‌های لازم در مورد بلوغ برای مادران و دختران نوجوان آن‌ها مفید می‌باشد و مانع بروز تعارض بین والدین با فرزندان و نیز کنترل عواطف در نوجوانان دختر می‌شود.

Title: تأثیر آموزش بر ارتقای رفتارهای پیشگیری‌کننده از سندرم پیش از قاعدگی در نوجوانان دختر: کاربرد الگوی اعتقاد بهداشتی

http://jech.umsha.ac.ir/article-1-253-fa.html

سابقه و هدف: سندرم پیش از قاعدگی بر کاهش کیفیت زندگی، عملکرد اجتماعی و تحصیلی نوجوانان و زنان جوان مؤثر است. نظر به اهمیت حفظ سلامتی دختران نوجوان، این پژوهش با هدف تعیین تأثیر آموزش مبتنی بر الگوی اعتقاد بهداشتی بر ارتقای رفتارهای پیشگیری‎کننده از سندرم پیش از قاعدگی در نوجوانان دختر اجرا شد. مواد و روش‌‌ها: این پژوهش نیمه‌تجربی در میان 120 نفر در قالب دو گروه 60 نفری آزمون و کنترل انجام شد و آزمودنی‌ها به شیوه نمونه‌گیری چند‌مرحله‌ای تصادفی انتخاب گردیدند. ابزار گردآوری داده‌ها شامل: پرسشنامه مشخصات دموگرافیک، پرسشنامه پژوهشگرساخته مشتمل‌بر آگاهی، رفتار و تمامی سازه‌های الگوی اعتقاد بهداشتی بود. مداخله آموزشی نیز 4 جلسه سخنرانی 60-45 دقیقه‌ای را شامل می‌شد. داده‌های گردآوری‌شده با استفاده از نرم‌افزارSPSS 16  و آزمون‌های Smirnov -Kolmogrov، Wilcoxon،T مستقل، T زوجی، Chi-Square و Mann–Whitney مورد تحلیل قرار گرفت. یافته‌ها: پیش از مداخله، تفاوت معناداری بین دو گروه از نظر متغیرهای دموگرافیکی و زمینه‌ای، آگاهی، رفتارهای پیشگیری‌کننده و تمامی اجزای الگو وجود نداشت (0.05P). نتیجه‌گیری: آموزش مبتنی بر الگوی اعتقاد بهداشتی در اتخاذ رفتارهای پیشگیرانه از سندرم پیش از قاعدگی مؤثر بود. با توجه به اهمیت سلامتی دختران به‌عنوان مادران آینده و کم‌هزینه‌بودن فعالیت‌های آموزش بهداشت نسبت به سایر فعالیت‌ها در این زمینه، لزوم بسط و تعمیم این‌گونه برنامه‌ها ضروری به‌نظر می‌رسد.

Title: تأثیر مداخله آموزشی مبتنی بر نظریه رفتار برنامه‌ریزی‌شده بر رفتار شیردهی زنان باردار شهر فسا

http://jech.umsha.ac.ir/article-1-358-fa.html

سابقه و هدف: با توجه به اهمیت تغذیه با شیر مادر و نقش عواملی چون نگرش و هنجارهای انتزاعی بر رفتار مادران و نیز میزان پایین تغذیه انحصاری با شیر مادر، پژوهش حاضر با هدف تعیین تأثیر برنامه آموزشی مبتنی بر نظریه رفتار برنامه‌ریزی‌شده بر رفتار شیردهی در زنان باردار شهر فسا انجام شد. مواد و روش‌‌ها: این پژوهش نیمه‌تجربی در سال 1396 بر روی 100 نفر از زنان باردار در سن بارداری 30 تا 34 هفته (50 نفر در گروه آزمون و 50 نفر در گروه کنترل) که به‌‌صورت تصادفی ساده از بین مراجعین مراکز بهداشتی- درمانی شهر فسا انتخاب شده بودند انجام شد. ابزار گردآوری اطلاعات شامل: پرسش‌نامه مشتمل بر اطلاعات جمعیت‌شناختی، سازه‌های نظریه رفتار برنامه‌ریزی‌شده و چک‌لیست بررسی تغذیه با شیر مادر بود. مداخله آموزشی به‌صورت 3 جلسه آموزشی 55 تا 60 دقیقه‌ای برای گروه آزمون برگزار گردید و رفتار شیردهی مادران قبل از آموزش و 40 روز پس از زایمان در هر دو گروه ارزیابی شد. لازم به ذکر است که اطلاعات با استفاده از نرم‌افزار 22 ‌SPSS و به‌کارگیری آزمون‌های کای اسکوئر، تی ‌مستقل و تی‌ زوجی تحلیل گردیدند. یافته‌ها: نتایج این مطالعه نشان می‌دهد که قبل از مداخله آموزشی اختلاف معنا‌داری بین دو گروه از نظر سازه‌های نظریه رفتار برنامه‌ریزی‌شده وجود ندارد (0.208=P)؛ اما پس از مداخله آموزشی در گروه آزمون تمام سازه‌های نظریه رفتار برنامه‌ریزی‌شده از جمله قصد و رفتار شیردهی بهبود یافته است (0.001=P). نتیجه‌گیری: براساس نتایج مشاهده شد که تغییر نگرش مادر نسبت به شیردهی با برنامه‌های آموزشی مناسب، حمایت شبکه‌های اجتماعی و وجود شرایط مناسب برای تغذیه با شیر مادر در جامعه از عوامل مؤثر در شیردهی موفق مادران در سه ماه اول پس از زایمان محسوب می‌شوند.
